# Dose-dependent effects of Nrf2 on the epidermis in chronic skin inflammation

**DOI:** 10.1242/dmm.052126

**Published:** 2025-01-02

**Authors:** Michael Koch, Luca Ferrarese, Maya Ben-Yehuda Greenwald, Sabine Werner

**Affiliations:** Institute of Molecular Health Sciences, Department of Biology, ETH Zürich, 8093 Zürich, Switzerland

**Keywords:** Skin, Atopic dermatitis, Keratinocyte, Nrf2, FGF, Cytoprotection

## Abstract

Atopic dermatitis (AD) is a chronic inflammatory skin disease, characterized by an impaired epidermal barrier and immunological alterations. The activity of the cytoprotective NRF2 transcription factor is reduced in the epidermis of AD patients. To determine the functional relevance of this deficiency, we used mice lacking fibroblast growth factor receptors 1 and 2 in keratinocytes (K5-R1/R2 mice), which exhibit several AD-like symptoms. Proteomics analysis of their epidermis revealed reduced Nrf2 activity. This was accompanied by an increase in DNA damage and in the number of senescent cells. Genetic deletion of *Nrf2* in keratinocytes of these mice further promoted DNA damage and senescence, but time-limited pharmacological activation of Nrf2 in the skin had a mild protective effect. Surprisingly, long-term genetic activation of Nrf2 in keratinocytes of K5-R1/R2 mice caused strong hyperkeratosis, keratinocyte hyperproliferation, epidermal thickening, increased keratinocyte apoptosis and DNA damage, and altered immune cell composition. These results reveal a complex role of Nrf2 in the epidermis and show the necessity to optimize the duration and intensity of NRF2 activation for the treatment of epidermal alterations in patients with AD.

## Teaser

**Summary:** Using a mouse model for the inflammatory skin disease atopic dermatitis, we show that appropriate activation of the cytoprotective Nrf2 transcription factor is of key relevance to epidermal homeostasis.

## INTRODUCTION

According to the Global Burden of Disease (GBD) study, skin diseases are the fourth most common disease type. Particularly common is atopic dermatitis (AD), a chronic, relapsing skin condition exhibiting a prevalence of 2-10% in adults and up to 20% in children (Weidinger et al., 2018). It is characterized by recurrent eczematous lesions and intense itch, frequently accompanied by erythroderma (Yew et al., 2019). Being multifactorial and highly heterogeneous between patients, AD is primarily driven by a combination of epidermal barrier defects and immunological alterations, promoting heightened type 2 responses and IgE-mediated sensitization to various allergens (Weidinger et al., 2018). Genetic predisposition and environmental influences determine the development of AD (Weidinger et al., 2018). Although immune dysregulation remains recognized as a key pathogenic factor, more recent studies identified epidermal barrier dysfunction as a crucial initiator of AD (Langan et al., 2020). The impaired barrier is caused by loss-of-function variants in genes that encode proteins required for barrier function, such as the cornified envelope protein filaggrin (Weidinger et al., 2006; Sandilands et al., 2007) or tight junction proteins (Katsarou et al., 2023; De Benedetto et al., 2011), or by reduced expression of these genes (Katsarou et al., 2023; Weidinger et al., 2018).

Several new and efficient AD therapeutics have recently entered the market (Schuler et al., 2023; Müller et al., 2024), but they mainly aim at symptom management. To develop alternative treatment regimens, it is crucial to investigate the cellular and molecular mechanisms underlying this skin condition. We recently discovered decreased activity of nuclear factor-erythroid 2-related factor 2 (NFE2L2; referred to here as NRF2) in the epidermis of lesional and, to a lesser extent, of non-lesional skin of AD patients using a discovery proteomics approach (Koch et al., 2023). NRF2 is a transcription factor that promotes the expression of genes involved in the detoxification of various compounds and of reactive oxygen species (ROS) (Hiebert and Werner, 2019; Hybertson et al., 2011). In the skin, Nrf2 protects from UV-induced apoptosis and controls metabolic and damage-related processes (Rojo de la Vega et al., 2018; Schäfer and Werner, 2015). Mice with global *Nrf2* knockout or conditional knockout of *Nrf2* in keratinocytes do not show obvious skin abnormalities under homeostatic conditions (Telorack et al., 2016; Braun et al., 2002). However, they had increased numbers of neutrophils in the skin on induction of contact dermatitis (Helou et al., 2019), and they developed more severe radiation-induced dermatitis (Schmidlin et al., 2020). Together, these findings suggest that activation of Nrf2 in the skin could be beneficial when the barrier function is impaired.

To study the effect of a gain of function of Nrf2, mice were generated that express a constitutively active mutant of Nrf2 in keratinocytes (K5-caNrf2 mice) (Schäfer et al., 2012). These mice exhibit acanthosis, hyperkeratosis and mild skin inflammation, resembling lamellar ichthyosis. Mechanistically, activation of Nrf2 promoted expression of the genes encoding the cornified envelope proteins small proline-rich protein (Sprr)2d and Sprr2h, which caused corneocyte fragility and disturbed desquamation, leading to impaired barrier function (Schäfer et al., 2012). However, activation of Nrf2 was beneficial when the barrier function was impaired, as shown by the rescue of the barrier function deficiency in loricrin knockout mice through activation of endogenous Nrf2 by compounds present in amniotic fluid (Huebner et al., 2012). Furthermore, expression of the *caNrf2* transgene partially rescued the epidermal defect in a mouse model for Netherton syndrome (Muzumdar et al., 2020). However, it is unclear whether and how alterations in the levels/activity of NRF2 affect an AD phenotype.

Although mouse models have obvious limitations for the study of human inflammatory skin disease because of the inherent differences between murine and human skin (Salgado et al., 2017), several murine models are available that show AD-like symptoms. One such model is mice lacking fibroblast growth factor receptor (Fgfr)1 and Fgfr2 in keratinocytes (K5-R1/R2 mice), which – like AD patients – exhibit increased transepidermal water loss (TEWL) and epidermal thickening, elevated serum IgE and IgG levels, and an increase in Langerhans cells, dermal T cells and mast cells (Yang et al., 2010; Sulcova et al., 2015). Furthermore, the gene expression pattern in the epidermis of these mice has similarities to the gene expression pattern in the epidermis of lesional AD skin (Ferrarese et al., 2024). The phenotype is, at least in part, a consequence of the strong downregulation of various tight junction proteins in the Fgfr-deficient keratinocytes (Yang et al., 2010) and of the loss of Fgfr2-mediated suppression of the expression of a battery of pro-inflammatory genes (Ferrarese et al., 2024). In addition to the AD-like symptoms, which result from the epidermal abnormalities, K5-R1/R2 mice develop progressive hair loss, because activation of Fgfr2 by Fgf7 and Fgf10 is required for hair follicle stem cell proliferation and thus for hair regeneration in mice (Greco et al., 2009; Yang et al., 2010).

Here, we used these K5-R1/R2 mice to study the effects of Nrf2 loss or gain of function in keratinocytes on the epidermal phenotype. Surprisingly, keratinocyte-specific loss of Nrf2 or its chronic activation both caused a more severe epidermal phenotype, although through different mechanisms. These results highlight the importance of appropriate NRF2 activation in the epidermis for the maintenance/restoration of epidermal barrier function in the context of inflammatory skin disease.

## RESULTS

### Progressive reduction of Nrf2 activity in keratinocytes, and increased DNA damage and senescence in K5-R1/R2 mice

To determine whether K5-R1/R2 mice are a suitable model to study the role of Nrf2 in the development of an AD-like phenotype, we further characterized their epidermal alterations. For this purpose, we re-analyzed a previously published proteomics dataset comparing the epidermis of adult K5-R1/R2 and control mice (Seltmann et al., 2018). The control mice were mice with floxed alleles, but without Cre expression (R1/R2 mice). These mice are suitable controls, because we had previously excluded effects of Cre in keratinocytes on the histological appearance of the epidermis and on DNA damage, proliferation and survival of keratinocytes, and expression of pro-inflammatory genes by these cells (Telorack et al., 2016; Yang et al., 2010). Furthermore, the use of floxed mice as control allowed for the comparison of mice from the same litter.

STRING analysis (Szklarczyk et al., 2019) revealed that K5-R1/R2 mice exhibit differences in the biological processes ‘establishment of skin barrier’, ‘keratinization’, ‘epidermal morphogenesis’ and ‘skin development’ (Fig. S1A). Proteins involved in these processes, which were more abundant in the epidermis of K5-R1/R2 mice, were keratin (Krt)14, Krt10 and Krt27, filaggrin-2, and late keratinocyte differentiation markers, such as Sprr4 or protein-glutamine gamma-glutamyltransferase 3 (Tgm3), which showed a high degree of clustering (Fig. S1B). Pathway analysis using ProteoRe and the Reactome databases (Mehta et al., 2023) revealed alterations in the inflammatory milieu, including significant activation of interleukin (IL)-1 signaling (Fig. S1C). Most of the differentially abundant proteins that belong to this pathway are involved in proteasomal degradation, while the cytokines themselves were not detected (Fig. S1D). As the proteomics analysis was performed on epidermal lysate, alterations in pro-inflammatory pathways are probably underestimated, because a majority of the cutaneous immune cells are located in the dermis.

**Fig. 1.**
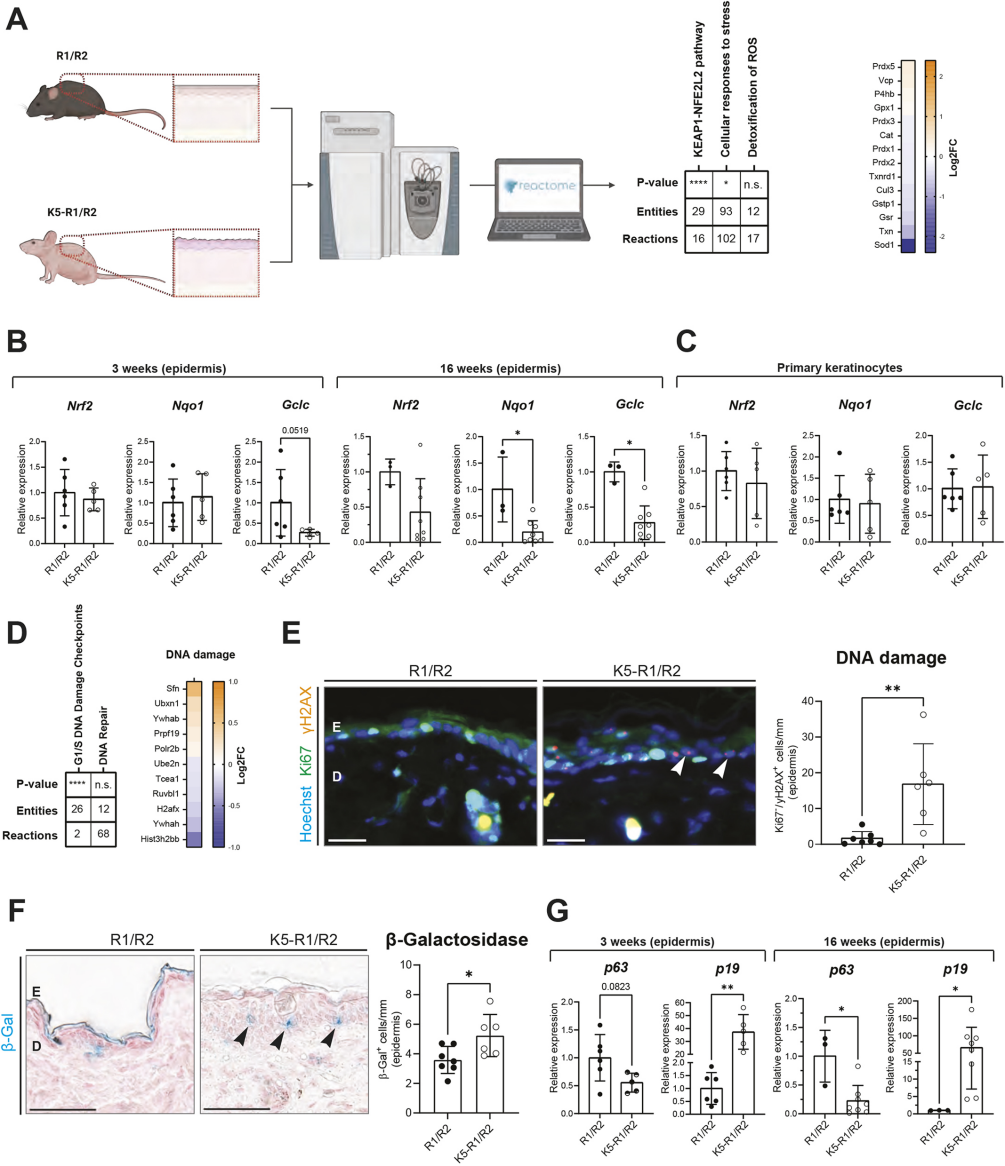
**Reduced Nrf2 activity, enhanced DNA damage and increased senescence in epidermal cells of K5-R1/R2 mice.** (A) Left: schematic representation of the mass spectrometry-based proteome analysis of the epidermis from K5-R1/R2 and control mice (R1/R2) (Seltmann et al., 2018). Created in BioRender by Werner, S. (2024). https://BioRender.com/s57n337. This figure was sublicensed under CC-BY 4.0 terms. Middle: table showing the regulation of oxidative stress response-related pathways. ROS, reactive oxygen species. Right: heat map showing differentially abundant proteins of these oxidative stress response-related pathways. Functional analysis was performed using ProteoRe. Log2FC, log2 fold change. (B) Reverse transcription quantitative PCR (RT-qPCR) for *Nrf2*, *Nqo1* and *Gclc* relative to *Rps29* using RNA from the epidermis of K5-R1/R2 and control mice at 3 (left) and 16 (right) weeks of age. *n*=3-8 mice per genotype. (C) RT-qPCR for *Nrf2*, *Nqo1* and *Gclc* relative to *Rps29* using RNA from primary murine keratinocytes of K5-R1/R2 and control mice. *n*=5-6 mice per genotype. (D) Left: table showing the regulation of DNA damage-related pathways in the epidermis of K5-R1/R2 *versus* control mice. Right: heat map showing individual differentially abundant proteins of these pathways. Functional analysis was performed using ProteoRe. (E) Left: representative images of immunofluorescence staining for γH2AX (red) and Ki67 (green), counterstained with Hoechst (blue) on back skin sections of control and K5-R1/R2 mice at the age of 12 weeks. Scale bars: 20 μm. Right: quantification of epidermal Ki67^−^/γH2AX^+^ keratinocytes (indicated by white arrowheads) in control and K5-R1/R2 mice. *n*=6-7 mice per genotype. D, dermis; E, epidermis. (F) Left: representative brightfield images of β-galactosidase (β-Gal) staining (blue) on back skin sections of control and K5-R1/R2 mice at the age of 12 weeks. Scale bars: 50 μm. Right: quantification of epidermal β-galactosidase^+^ keratinocytes (indicated by black arrowheads). Note the unspecific staining of the stratum corneum. *n*=6-7 mice per genotype. (G) RT-qPCR for *p63* and *p19* relative to *Rps29* using RNA from the epidermis of K5-R1/R2 and control mice at 3 (left) or 16 (right) weeks of age. *n*=3-8 mice per genotype and time point. Graphs indicate mean±s.d. n.s., non-significant; *P<0.05, **P<0.01, ****P<0.0001 (Mann–Whitney *U*-test).

Interestingly, there was a highly significant reduction in the abundance of components of the Nrf2 pathway (referred to here as ‘KEAP1-NFE2L2 pathway’) in the epidermis of K5-R1/R2 mice, as well as changes in ‘cellular responses to stress’ and mild, although non-significant, changes in ‘detoxification of ROS’ (Fig. 1A). To verify these findings, we performed reverse transcription quantitative PCR (RT-qPCR) for *Nrf2* and Nrf2 transcriptional target genes. In the epidermis of 3-week-old K5-R1/R2 mice, we only detected a mild reduction in *Gclc* expression compared to that in the epidermis of age-matched control mice, while expression of *Nrf2* itself and of its major target NAD(P)H quinone dehydrogenase (*Nqo1*) (McMahon et al., 2001) were not affected (Fig. 1B). At 16 weeks, however, when the inflammatory and epidermal phenotype was fully developed (Yang et al., 2010), expression of *Nqo1* and *Gclc* was strongly downregulated (Fig. 1B), and a mild, although non-significant, reduction in the expression of *Nrf2* itself was also detected. Expression of Nrf2 target genes was not reduced in cultured primary keratinocytes from neonatal K5-R1/R2 mice (Fig. 1C). This finding, and the normal expression/activity of Nrf2 in the epidermis of young (3-week-old) K5-R1/R2 mice *in vivo*, suggests that reduced Nrf2 activation is not a direct consequence of the loss of *Fgfr1* and *Fgfr2*, but possibly results from the chronic inflammation and/or the impaired epidermal barrier.

Together, these findings demonstrate that – similar to AD in humans – K5-R1/R2 epidermis exhibits decreased activity of Nrf2, which progresses with the age-dependent development of the inflammatory skin phenotype.

### Loss of Nrf2 promotes DNA damage and senescence of keratinocytes *in vivo*

Pathway analysis using the proteomics dataset from the epidermis of K5-R1/R2 and control mice revealed a highly significant change in ‘G1/S DNA damage checkpoints’ and mild alterations in ‘DNA repair’, indicating activation of the DNA damage response (Fig. 1D). Staining for γH2AX, a histone modification only present upon DNA damage as well as in proliferating cells, where it is present in combination with the cell proliferation marker Ki67 (also known as Mki67) (Kuo and Yang, 2008; Rogakou et al., 1998), identified a significantly increased percentage of cells positive for γH2AX and negative for Ki67 compared to that in control mice at the age of 12 weeks, demonstrating an increase in cells with DNA damage (Fig. 1E). This is consistent with previously observed oxidative stress and DNA damage in AD patients (Khan et al., 2022; Blunder et al., 2018). Because DNA damage frequently causes cellular senescence (d'Adda di Fagagna, 2008), we stained the skin for β-galactosidase activity, a characteristic marker for senescent cells (Valieva et al., 2022; Dimri et al., 1995). There was a significant increase in β-galactosidase-positive keratinocytes in the epidermis of K5-R1/R2 mice at 12 weeks of age (Fig. 1F). Expression of the senescence marker *p19* (also known as *Cdkn2d*) was already strongly increased at 3 weeks of age (Fig. 1G). Concomitantly, *p63* (also known as *Trp63*), the loss of which in keratinocytes induces senescence and which is downregulated in senescent keratinocytes (Keyes et al., 2005; Piccinni et al., 2013), was mildly, although non-significantly, downregulated at this time point. The alterations in *p19* and *p63* expression were more pronounced at 16 weeks (Fig. 1G). These findings demonstrate that K5-R1/R2 mice have increased numbers of keratinocytes that exhibit DNA damage and signs of cellular senescence, a phenotype that progresses with age.

### Loss of Nrf2 promotes DNA damage and senescence in K5-R1/R2 mice

**Fig. 2.**
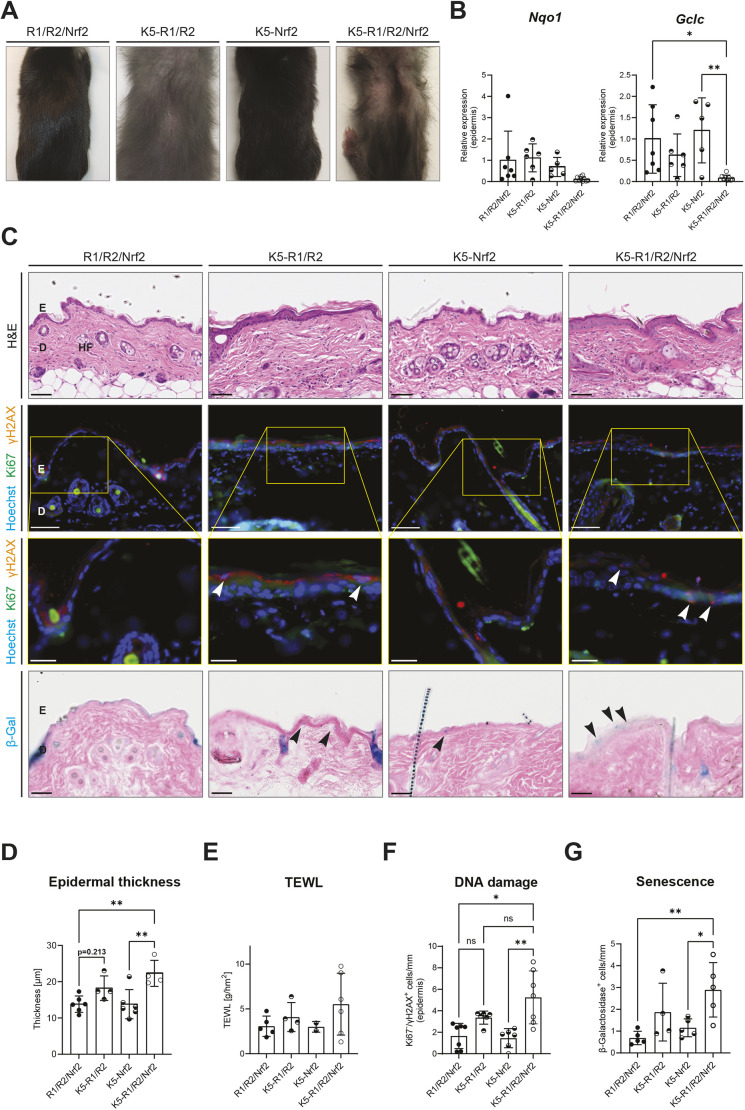
**Loss of Nrf2 in keratinocytes of K5-R1/R2 mice mildly aggravates their epidermal phenotype.** (A) Representative macroscopic images of the back of control (R1/R2/Nrf2), K5-R1/R2, K5-Nrf2 and K5-R1/R2/Nrf2 mice (left to right). (B) RT-qPCR for *Nqo1* and *Gclc* relative to *Rps29* using RNA from the epidermis of control, K5-R1/R2, K5-Nrf2 and K5-R1/R2/Nrf2 mice at 6 weeks of age. *n*=5-10 mice per genotype. (C) Representative brightfield photomicrographs of H&E staining (top row); immunofluorescence for γH2AX (red) and Ki67 (green), counterstained with Hoechst (blue) (second and third rows); and brightfield photomicrographs of β-galactosidase staining (bottom row) on back skin sections of control, K5-R1/R2, K5-Nrf2 and K5-R1/R2/Nrf2 mice at 6 weeks of age. Scale bars: 50 μm (top, second and bottom rows) and 20 μm (third row). D, dermis; E, epidermis; HF, hair follicle. The area outlined by a yellow rectangle in the second row is shown at higher magnification in the third row. Ki67^−^/γH2AX^+^ keratinocytes are indicated by white arrowheads; β-galactosidase^+^ keratinocytes are indicated by black arrowheads. (D) Quantification of epidermal thickness based on H&E staining (C) of control, K5-R1/R2, K5-Nrf2 and K5-R1/R2/Nrf2 mice at 6 weeks of age. *n*=4-6 mice per genotype. (E) Absolute values of transepidermal water loss (TEWL) in control, K5-R1/R2, K5-Nrf2 and K5-R1/R2/Nrf2 mice at 6 weeks of age. *n*=2-6 mice per genotype. (F) Quantification of epidermal Ki67^−^/γH2AX^+^ keratinocytes in control, K5-R1/R2, K5-Nrf2 and K5-R1/R2/Nrf2 mice at 6 weeks of age. *n*=6-7 mice per genotype. (G) Quantification of epidermal β-galactosidase^+^ keratinocytes in control, K5-R1/R2, K5-Nrf2 and K5-R1/R2/Nrf2 mice at 6 weeks of age. *n*=4-5 mice per genotype. Graphs indicate mean±s.d. n.s., non-significant; *P<0.05, **P<0.01 (one-way ANOVA).

To determine whether the remaining Nrf2 activity in keratinocytes of K5-R1/R2 mice prevents an even more severe phenotype, we generated triple knockout mice lacking functional *Fgfr1*, *Fgfr2* and *Nrf2* genes in keratinocytes (K5-R1/R2/Nrf2 mice). Similar to K5-R1/R2 mice (Yang et al., 2010), the triple knockout mice showed progressive hair loss (Fig. 2A). They also had a lower body weight (K5-R1/R2 mice, 13.7±3.4 g; K5-R1/R2/Nrf2 mice, 11.8±2.4 g) than that of control mice (R1/R2/Nrf2 mice) at the age of 6 weeks, although the difference was not statistically significant. Some of the triple mutant mice were even smaller than K5-R1/R2 mice and appeared fragile, and thus had to be sacrificed for animal welfare reasons. Mice exhibiting only loss of *Nrf2* in keratinocytes (K5-Nrf2 mice) were macroscopically comparable to floxed control mice (Fgfr1/Fgfr2/Nrf2 mice) (Fig. 2A) (Telorack et al., 2016).

Because of the fragility of the triple mutant mice, all further analyses were performed at the age of 6 weeks. Expression of the Nrf2 target gene *Nqo1* was moderately, but non-significantly, downregulated in K5-R1/R2/Nrf2 mice at this time point. The triple mutant mice also exhibited downregulation of *Gclc*, whereas expression of this Nrf2 target was unchanged in K5-Nrf2 mice (Fig. 2B).

The skin of 6-week-old K5-R1/R2/Nrf2 mice showed similar histopathological features to those of K5-R1/R2 mice (Fig. 2C, top row), including epidermal thickening, whereas K5-Nrf2 mice did not show obvious histological abnormalities (Fig. 2C, top row; Fig. 2D). There was also no significant increase in the inside-out epidermal barrier between K5-R1/R2 and K5-R1/R2/Nrf2 mice as determined by measuring TEWL (Fig. 2E).

Staining for γH2AX revealed a mild increase in the number of keratinocytes with DNA damage (Ki67^−^/γH2AX^+^) in K5-R1/R2/Nrf2 versus K5-R1/R2 mice, but the difference was not statistically significant owing to the high variability in the triple mutant mice (Fig. 2C, second and third rows; Fig. 2F). The increase in DNA damage in K5-R1/R2 mice was less pronounced at this age compared to that in adult mice (see Fig. 1E).

β-Galactosidase staining showed a mild, but non-significant, increase in the number of senescent epidermal cells of K5-R1/R2/Nrf2 mice compared to that of K5-R1/R2 mice. Loss of Nrf2 alone did not promote cellular senescence (Fig. 2C, bottom row; Fig. 2G). Consistent with our previous data (Fig. 1G), expression of *p19* was significantly increased in K5-R1/R2 compared to that in control mice. However, the upregulation was less pronounced in K5-R1/R2/Nrf2 mice. K5-Nrf2 mice did not show altered *p19* expression (Fig. S2A). Expression of *p63* was similar in mice of all genotypes (Fig. S2A).

Taken together, these results indicated that the loss of Nrf2 only mildly aggravated the epidermal phenotype of K5-R1/R2 mice.

### Pharmacological activation of Nrf2 promotes barrier function in K5-R1/R2 mice

**Fig. 3.**
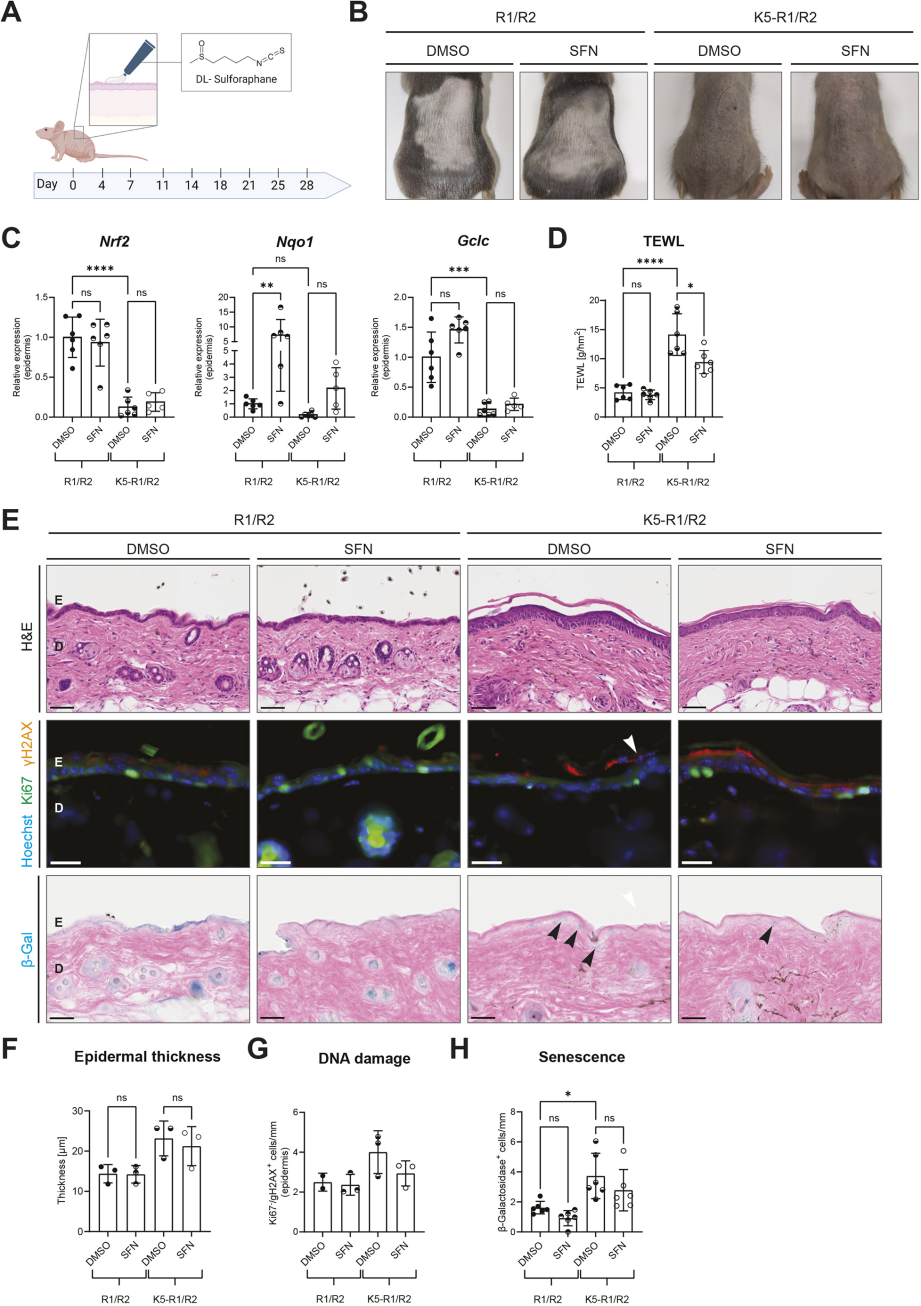
**Pharmacological activation of Nrf2 in keratinocytes of K5-R1/2 mice promotes epidermal barrier function.** (A) Schematic representation of the topical pharmacological treatment of control (R1/R2) and K5-R1/R2 mice with sulforaphane (SFN) or vehicle. The treatment regimen and the chemical formula of SFN are shown. Created in BioRender by Werner, S. (2024). https://BioRender.com/h63i066. This figure was sublicensed under CC-BY 4.0 terms. (B) Representative macroscopic images of the back of control and K5-R1/R2 mice after 4 weeks of topical treatment with SFN or vehicle. (C) RT-qPCR for *Nrf2*, *Nqo1* and *Gclc* relative to *Rps29* using RNA from the epidermis of control and K5-R1/R2 mice after a 4-week treatment with SFN or vehicle. *n*=5-6 mice per genotype and treatment group. (D) Absolute values of TEWL in control and K5-R1/R2 mice after a 4-week treatment with SFN or vehicle. *n*=6 mice per genotype and treatment group. (E) Representative brightfield images of H&E staining (top row); immunofluorescence for γH2AX (red) and Ki67 (green), counterstained with Hoechst (blue) (middle row); and brightfield images of β-galactosidase staining (bottom row) on back skin sections of adult control and K5-R1/R2 mice after a 4-week topical treatment with SFN or vehicle. Scale bars: 50 μm. Ki67^−^/γH2AX^+^ or β-galactosidase^+^ keratinocytes are indicated by white or black arrowheads, respectively. D, dermis; E, epidermis. (F) Epidermal thickness based on H&E staining (E) of back skin sections from control and K5-R1/R2 mice after a 4-week treatment with SFN or vehicle. *n*=3 mice per genotype and treatment group. (G) Quantification of epidermal Ki67^−^/γH2AX^+^ keratinocytes in control and K5-R1/R2 mice after a 4-week treatment with SFN or vehicle. *n*=2-3 mice per genotype and treatment group. (H) Quantification of epidermal β-galactosidase^+^ keratinocytes in control and K5-R1/R2 mice after a 4-week treatment with SFN or vehicle. *n*=6 mice per genotype and treatment group. C, D and H show combined data of two independent experiments. Graphs indicate mean±s.d. n.s., non-significant; *P<0.05, **P<0.01, ***P<0.001, ****P<0.0001 (two-way ANOVA).

We next tested whether activation of Nrf2 ameliorates the epidermal abnormalities of K5-R1/R2 mice. Therefore, we attempted to activate endogenous Nrf2 in the skin of 12-week-old K5-R1/R2 mice through topical application of sulforaphane (SFN). SFN is a well-established Nrf2-activating compound with low toxicity (Zhou et al., 2016), which has been used for topical application in other settings (Schäfer et al., 2012; Kerns et al., 2010). The shaved back skin of control and K5-R1/R2 mice was topically treated twice per week for 4 weeks with a hydrophilic, non-ionic cream containing either SFN in dimethyl sulfoxide (DMSO; vehicle) or vehicle only (Fig. 3A). This procedure allows the penetration of Nrf2-activating compounds into the epidermis and the dermis, thereby activating all skin cell types (Durchdewald et al., 2007). A 4-week treatment with SFN did not induce visible alterations in K5-R1/R2 or control mice (Fig. 3B). It did not affect expression of *Nrf2* itself, but caused a strong and significant increase in the expression of *Nqo1* in control mice. However, expression of this gene was only mildly, but non-significantly, increased in K5-R1/R2 mice (Fig. 3C), and expression levels of *Gclc* remained unchanged. These findings demonstrate that the basal, as well as the inducible, activity of Nrf2 is reduced in K5-R1/R2 mice. Nevertheless, SFN treatment partially reverted the increased TEWL observed in K5-R1/R2 mice (Yang et al., 2010), suggesting improvement of epidermal barrier function (Fig. 3D).

There were no obvious histological differences between vehicle- and SFN-treated control mice (Fig. 3E, top row). The epidermal thickness was increased in K5-R1/R2 mice as described previously (Yang et al., 2010). SFN treatment did not affect the epidermal thickness in either K5-R1/R2 or control mice (Fig. 3E, top row; Fig. 3F).

DNA damage and senescence were not affected by SFN treatment in control mice, and the compound only had a very mild, and non-significant, effect on these parameters in K5-R1/R2 mice. The increased percentage of Ki67^−^/γH2AX^+^ and of β-galactosidase-positive keratinocytes that we observed in untreated or vehicle-treated K5-R1/R2 mice (Fig. 3E, second and third rows; Fig. 3G,H) was moderately, although non-significantly, reduced by SFN treatment. This was confirmed in an independent experiment (Fig. S2B).

Together, these findings demonstrate that transient activation of Nrf2 in K5-R1/R2 mice by SFN improves their epidermal barrier. However, the cytoprotective effect was minor, most likely because of the limited and short-term activation of Nrf2 that was achieved in these mice.

### Genetic activation of Nrf2 in K5-R1/R2 mice causes strong hyperkeratosis

**Fig. 4.**
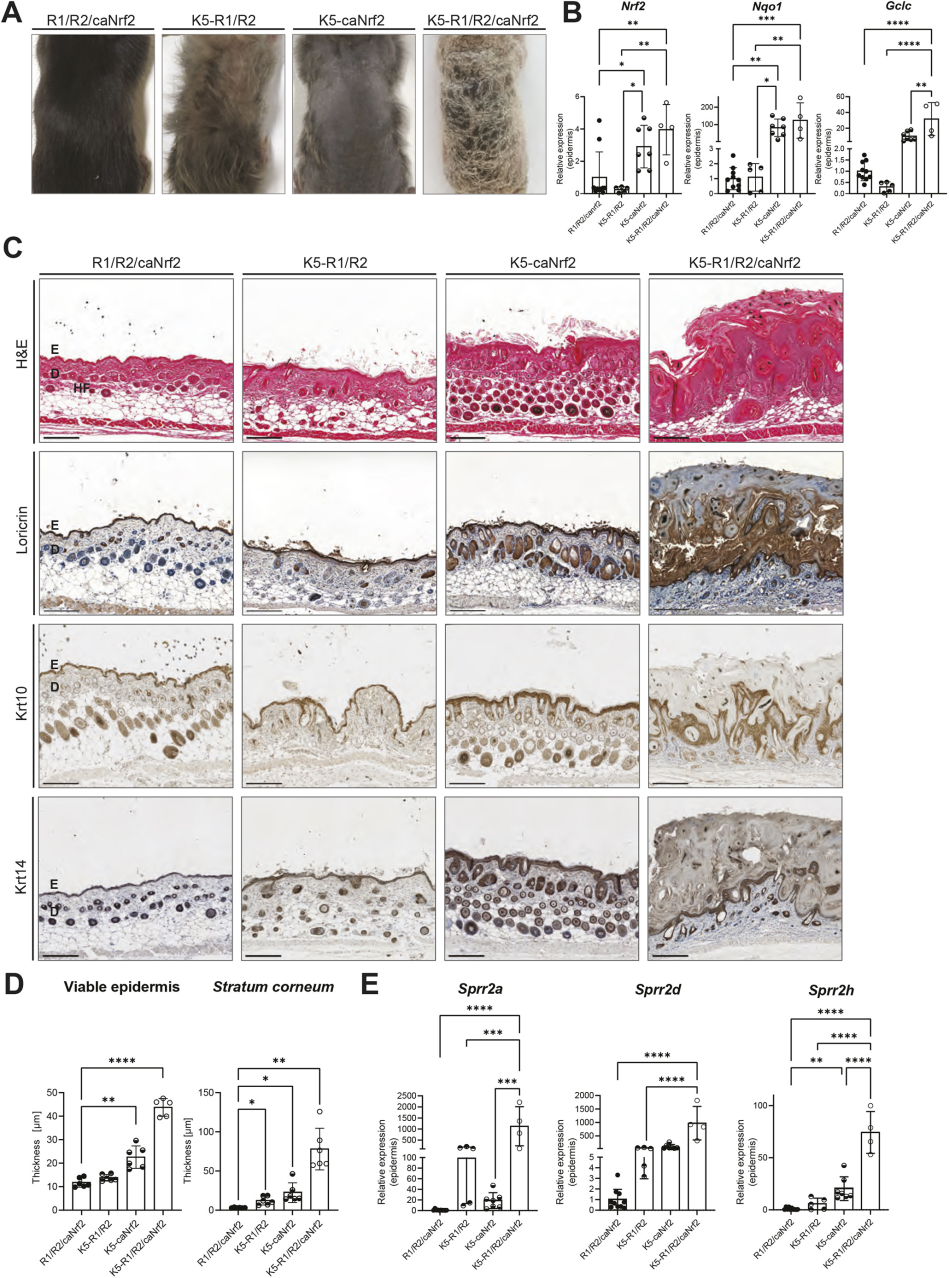
**Constitutive activation of Nrf2 in keratinocytes of K5-R1/R2 mice causes strong hyperkeratosis.** (A) Representative macroscopic images of the back of control (R1/R2/caNrf2), K5-R1/R2, K5-caNrf2 and K5-R1/R2/caNrf2 mice. (B) RT-qPCR for *Nrf2*, *Nqo1* and *Gclc* relative to *Rps29* using RNA from the epidermis of control, K5-R1/R2, K5-caNrf2 and K5-R1/R2/caNrf2 mice at 3 weeks of age. *n*=4-10 mice per genotype. (C) Representative brightfield images of H&E staining (top row), and immunohistochemical staining of loricrin (second row), Krt10 (third row) and Krt14 (fourth row), counterstained with Hematoxylin, on back skin sections of control, K5-R1/R2, K5-caNrf2 and K5-R1/R2/caNrf2 mice at 3 weeks of age. Scale bars: 100 μm. D, dermis; E, epidermis; HF, hair follicle. (D) Thickness of the viable epidermis (left) and the stratum corneum (right) based on H&E staining (C) of back skin sections from control, K5-R1/R2, K5-caNrf2 and K5-R1/R2/caNrf2 mice at 3 weeks of age. *n*=5-6 mice per genotype. (E) RT-qPCR for *Sprr2a*, *Sprr2d* and *Sprr2h* relative to *Rps29* using RNA from the epidermis of control, K5-R1/R2, K5-caNrf2 and K5-R1/R2/caNrf2 mice at 3 weeks of age. *n*=4-10 mice per genotype. Graphs indicate mean±s.d. n.s., non-significant; *P<0.05, **P<0.01, ***P<0.001, ****P<0.0001 (one-way ANOVA).

Because of the poor activation of Nrf2 that was achieved by topical SFN treatment, we next tested whether long-term genetic activation of Nrf2 in keratinocytes of K5-R1/R2 mice has a greater beneficial effect. Therefore, we crossed K5-R1/R2 mice with mice expressing a constitutively active Nrf2 mutant (caNrf2) in a Cre-dependent manner. In these mice, the *caNrf2* transgene is under control of a constitutively active β-actin promoter and a CMV enhancer and preceded by a floxed STOP cassette. In the presence of Cre, the STOP cassette is excised and *caNrf2* is expressed (Schäfer et al., 2012). When crossed with K5-Cre mice, the transgene is expressed in all keratinocytes, starting during late embryonic development (Schäfer et al., 2012). In contrast to the topical application of SFN, other cell types are not affected with this approach. In a previous study, the extent of Nrf2 activation in these mice was comparable to the activation level that was achieved upon daily treatment of wild-type mice with the Nrf2-activating compound tert-butylhydroquinone (Schäfer et al., 2012), but it was higher than that in SFN-treated mice (Schäfer et al., 2012; this study). K5-caNrf2 mice are smaller than control mice and exhibit mild hyperkeratosis, acanthosis, and abnormalities of the sebaceous glands and hair follicles (Schäfer et al., 2012). Interestingly, K5-R1/R2 caNrf2 mice had a further decrease in body weight compared to that of K5-R1/R2 or K5-caNrf2 mice at 3 weeks of age (Fig. S2C). Remarkably, they showed strong hyperkeratosis over the entire body (Fig. 4A); therefore, we did not analyze more mice at this or later stages, but sacrificed the mice at the onset of the hyperkeratosis for animal welfare reasons. As expected, expression of *Nrf2/caNrf2*, *Nqo1* and *Gclc* was significantly upregulated in K5-R1/R2/caNrf2 compared to that in control mice that lack the K5-Cre transgene (R1/R2/caNrf2 mice) or K5-R1/R2 mice at this age, but it was similar to that in K5-caNrf2 mice (Fig. 4B). Hematoxylin and Eosin (H&E) staining confirmed the hyperkeratosis and revealed a severely thickened viable epidermis and, in particular, a much thicker stratum corneum (Fig. 4C, top row; Fig. 4D). Both parts of the epidermis were already enlarged in K5-caNrf2 mice, as described previously (Schäfer et al., 2012), but the phenotype was strongly exacerbated in K5-R1/R2/caNrf2 mice. Furthermore, their epidermis appeared highly disorganized. Loricrin, a marker of terminal differentiation, was restricted to the granular and cornified layers in mice of all genotypes, but the thickness of the loricrin-positive part of the epidermis was strongly increased in K5-R1/R2/caNrf2 mice. Interestingly, the uppermost layer of the stratum corneum was loricrin negative (Fig. 4C, second row). Krt10, which is normally expressed in suprabasal keratinocytes, but absent in the stratum basale and stratum corneum, was not consistently present in the lower suprabasal layers of K5-R1/R2/caNrf2 mice (Fig. 4C, third row). Krt14 expression was restricted to the stratum basale in mice of all genotypes. However, the Krt14-positive layer appeared thicker and much more irregular in K5-R1/R2/caNrf2 mice than in the other mouse groups (Fig. 4C, fourth row).

K5-caNrf2 mice were previously shown to overexpress the cornified envelope proteins Sprr2d and Sprr2h, leading to corneocyte fragility in adult mice (Schäfer et al., 2012). Expression of *Sprr2a*, *Sprr2d* and *Sprr2h* was also increased in K5-R1/R2 and K5-caNrf2 mice at postnatal day (P)18 and reached highest levels in K5-R1/R2/caNrf2 mice (Fig. 4E). This may further affect the cornified envelope and result in a compensatory increase in the thickness of the stratum corneum. Notably, TEWL was not altered between mice of different genotypes at the neonatal stage (Fig. S3A). This is consistent with the normal TEWL in K5-R1/R2 mice at P18, but its strong increase in adult mice of this genotype (Yang et al., 2010). The stability of the corneocytes was also similar in neonatal mice of all genotypes (Fig. S3B), suggesting that the phenotype observed in K5-R1/R2/caNrf2 mice develops progressively. We could not analyze TEWL or corneocyte stability in K5-R1/R2/caNrf2 mice at P18 or later stages, because their severe phenotype did not allow us to generate and analyze additional animals required for these experiments.

**Fig. 5.**
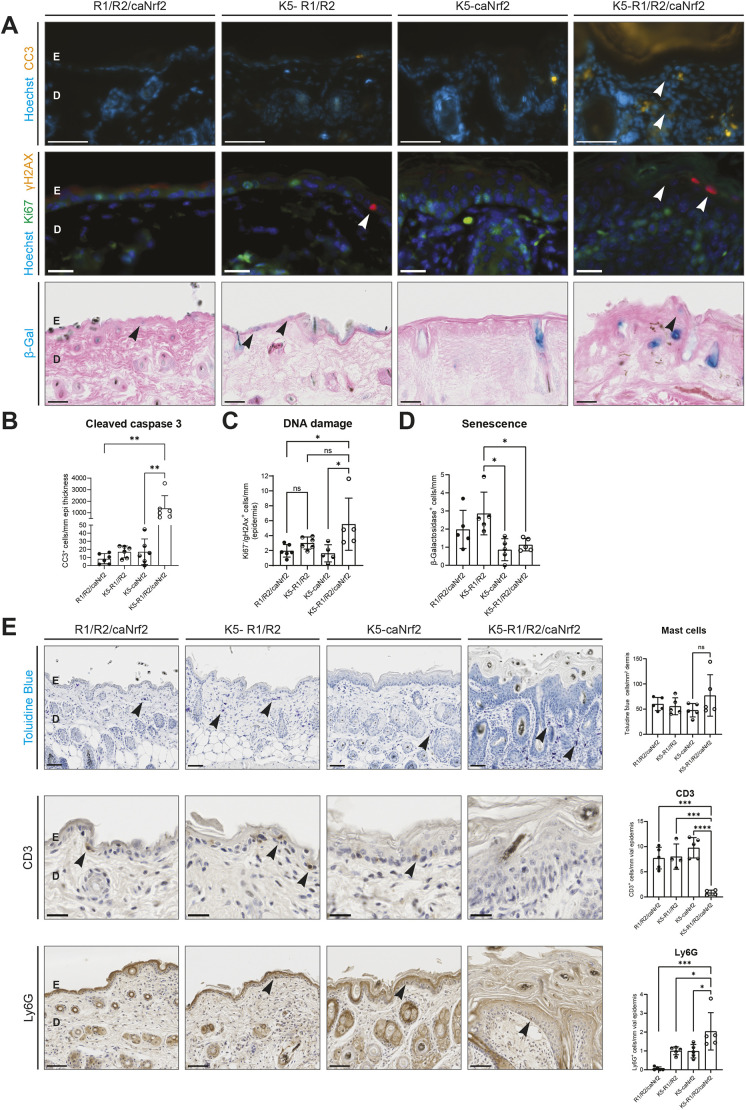
**Constitutive activation of Nrf2 in keratinocytes of K5-R1/R2 mice promotes apoptosis and alters the immune cell composition.** (A) Representative images of immunofluorescence for cleaved caspase 3 (CC3; red, top row), or Ki67 (green) combined with γH2AX (red) (middle row), counterstained with Hoechst (blue) on back skin sections of control (R1/R2/caNrf2), K5-R1/R2, K5-caNrf2 and K5-R1/R2/caNrf2 mice at 3 weeks of age. Apoptotic keratinocytes or keratinocytes with DNA damage are indicated with white arrowheads. Scale bars: 50 μm (top row) or 20 μm (middle row). Bottom row shows brightfield images of β-galactosidase staining on back skin sections of mice of all genotypes at 3 weeks of age. β-galactosidase^+^ keratinocytes are indicated with black arrowheads. Scale bars: 50 μm. (B) Quantification of epidermal cleaved caspase 3-positive keratinocytes of control, K5-R1/R2, K5-caNrf2 and K5-R1/R2/caNrf2 mice as shown in A. *n*=6 mice per genotype. (C) Quantification of epidermal Ki67^−^/γH2AX^+^ keratinocytes of control, K5-R1/R2, K5-caNrf2 and K5-R1/R2/caNrf2 mice as shown in A. *n*=5-6 mice per genotype. (D) Quantification of epidermal β-galactosidase^+^ keratinocytes of control, K5-R1/R2, K5-caNrf2 and K5-R1/R2/caNrf2 mice as shown in A. *n*=5 mice per genotype. (E) Representative brightfield images of Toluidine Blue staining for mast cells (top row) and immunohistochemical staining of CD3 (middle row) and Ly6G (bottom row) on back skin sections of control (R1/R2/caNrf2), K5-R1/R2, K5-caNrf2 and K5-R1/R2/caNrf2 mice at 3 weeks of age. Mast cells (top row), epidermal CD3^+^ T cells (middle row) and epidermal Ly6G^+^ neutrophils (bottom row) are indicated by black arrowheads. Graphs show quantification of Toluidine Blue-positive dermal cells (top), CD3^+^ epidermal cells (middle) and Ly6G^+^ epidermal cells (bottom). Scale bars: 50 μm (top and bottom rows) or 20 μm (middle row). *n*=5 mice per genotype. D, dermis, E, epidermis. Graphs indicate mean±s.d. n.s., non-significant; *P<0.05, **P<0.01, ***P<0.001, ****P<0.0001 (one-way ANOVA).

We next investigated whether the severely disturbed epidermal homeostasis in the triple mutant mice at P18 is accompanied by increased keratinocyte apoptosis. Consistent with our previous findings (Yang et al., 2010), the number of cleaved caspase 3-positive (apoptotic) keratinocytes was equally low in the epidermis of control and K5-R1/R2 mice, whereas K5-R1/R2/caNrf2, but not K5-caNrf2 mice, exhibited a strong and significant increase in the number of apoptotic keratinocytes (Fig. 5A, top row; Fig. 5B). There was also a mild, but non-significant, increase in Ki67^−^/γH2AX^+^ epidermal cells in K5-R1/R2/caNrf2 mice at P18, suggesting a moderate increase in DNA damage at this early stage (Fig. 5A, second row; Fig. 5C). However, the number of epidermal β-galactosidase-positive cells was even reduced in K5-caNrf2 and K5-R1/R2/caNrf2 mice (Fig. 5A, third row; Fig. 5D), possibly because of a higher epidermal turnover.

Last, we investigated whether the disturbed epidermal barrier affects immune cells, which we previously showed to be increased in K5-R1/R2 mice (Sulcova et al., 2015; Yang et al., 2010). Analysis of mast cells by Toluidine Blue staining revealed no obvious differences between genotypes (Fig. 5E, top tow). CD3^+^ T cells were slightly, although non-significantly, increased in the epidermis of K5-caNrf2 mice. Surprisingly, K5-R1/R2/caNrf2 mice exhibited a strong reduction in epidermal T cells, which may also result from a faster epidermal turnover (Fig. 5E, middle row). Ly6G^+^ neutrophils, which are major ROS-producing immune cells (Winterbourn et al., 2016), were almost absent in control mice and non-significantly increased in the epidermis of K5-R1/R2 and K5-caNrf2 mice (Fig. 5E, bottom row), as previously described (Yang et al., 2010; Schäfer et al., 2012). K5-R1/R2/caNrf2 mice showed an even further, although moderate, increase in epidermal neutrophils (Fig. 5E, bottom row).

Taken together, these results indicate that K5-R1/R2/caNrf2 mice did not show attenuation of the epidermal phenotype observed in K5-R1/R2 mice. Instead, they developed severe and progressive hyperkeratosis and disturbed epidermal differentiation, accompanied by increased keratinocyte DNA damage, apoptosis, a reduction in epidermal T cells and an increase in epidermal neutrophils.

## DISCUSSION

In this study, we discovered dose-dependent effects of Nrf2 on the epidermis in a mouse model of chronic skin inflammation (K5-R1/R2 mice). Consistent with human data obtained in a proteomics study of isolated epidermis from lesional and non-lesional skin of ten AD patients and ten healthy volunteers (Koch et al., 2023), these mice exhibited reduced Nrf2 activity in the epidermis. This finding correlates with the broad variety of AD-like symptoms that occur in these mice (Sulcova et al., 2015), suggesting them as a suitable model to study the role of different genes in the development of an AD-like epidermal phenotype. The reduced activity of NRF2 that we observed in AD patients as well as in our mouse model is consistent with the reduced expression of the NRF2 target glutathione S-transferase A4 (GSTA4) in lesional skin of AD patients (Ma et al., 2024).

K5-R1/R2 mice also had an increased number of epidermal cells with DNA damage and more senescent cells. These abnormalities progressed with age, suggesting that they are a consequence of the progressive deficiency in epidermal barrier function. Owing to technical limitations, we were so far unable to determine whether they also exhibit increased levels of ROS in the epidermis *in vivo*, which may contribute to the DNA damage and senescence, because ROS directly promote cellular senescence by various mechanisms (Davalli et al., 2016; van Deursen, 2014). However, we previously showed that knockdown of NRF2 in cultured human keratinocytes increases the levels of ROS (Koch et al., 2023). Therefore, it is likely that reduced Nrf2 activity leads to accumulation of ROS and consequent cellular damage in K5-R1/R2 mice. This is consistent with the proposed key role of oxidative stress in AD pathogenesis (Bertino et al., 2020).

The loss of Nrf2 mildly aggravated the epidermal phenotype of K5-R1/R2 mice. This included a further increase in senescent cells, while the percentage of epidermal cells with DNA damage was similar to that in the *Nrf2* single knockout or control mice. Therefore, the remaining mild Nrf2 activity in keratinocytes of K5-R1/R2 mice does not provide significant cytoprotection in the epidermis. It remains to be determined whether it protects the epithelium in the oral cavity or upper esophagus, which could explain the enhanced frailty of the triple knockout mice.

Interestingly, a normal activity of Nrf2 in keratinocytes was previously shown to protect from skin inflammation in other settings. Although mice with global *Nrf2* knockout do not exhibit skin abnormalities under homeostatic conditions, they showed an increase in neutrophils in the epidermis due to increased oxidative stress and enhanced chemokine production upon induction of contact dermatitis (Helou et al., 2019). Furthermore, *Nrf*2 knockout mice developed more severe radiation-induced dermatitis than did control mice (Schmidlin et al., 2020).

To investigate whether the cytoprotective effect of Nrf2 activation can be exploited in an AD-like setting, we pharmacologically activated endogenous Nrf2 in the skin by topical SFN treatment. This treatment did not have obvious adverse effects and even improved the inside-out barrier of the epidermis in K5-R1/R2 mice. However, the cytoprotective effect was marginal, most likely because of the generally limited activity of Nrf2 in the epidermis of K5-R1/R2 that we discovered in this study. Therefore, it may be necessary to use higher concentrations of SFN, longer or more frequent treatment, or systemic application.

In contrast to the weak effect in K5-R1/R2 mice, Nrf2-activating compounds were protective in other mouse models for inflammatory skin disease, in which a more potent activation of endogenous Nrf2 could be achieved. For example, systemic SFN treatment reduced mast cell infiltration, eosinophil count and serum IgE levels in a 2,4-dinitrochlorobenzene (DNCB)-induced AD mouse model (Wu et al., 2019), suggesting that Nrf2 activation also attenuates the inflammatory response. In another study, the Nrf2-activating compound Ku-Gan ameliorated the skin lesions in a mouse model of recurrent AD, which mainly involved increased expression of the Nrf2 target Gsta4 (Ma et al., 2024). Activation of Nrf2 by topical treatment with apocarotenoid mitigated radiation-induced dermatitis in mice (Schmidlin et al., 2020), and cardamonin treatment activated Nrf2 in an oxazolone-induced AD mouse model, which suppressed Th2 cytokine production and protected from oxidative DNA and lipid damage (Yoo et al., 2020). However, Nrf2-independent effects of these compounds cannot be fully excluded, as previously shown for SFN (Kerns et al., 2010).

Because of the limited basal and inducible activity of Nrf2 in the epidermis of K5-R1/R2 mice, we generated K5-R1/R2/caNrf2 mice. Surprisingly, these mice developed much more severe hyperkeratosis than K5-caNrf2 mice, combined with major defects in keratinocyte differentiation. However, they developed very few cysts, a hallmark of K5-caNrf2 mice (Schäfer et al., 2014), most likely because of the lack of hair follicles that require Fgfr1 and Fgfr2 for their regeneration (Yang et al., 2010). The massive hyperkeratosis in K5-R1/R2/caNrf2 mice was unexpected and likely resulted from a combination of alterations that affect the epidermal barrier. K5-caNrf2 mice exhibit increased expression of *Sprr2d* and *Sprr2h*, ultimately leading to corneocyte fragility (Schäfer et al., 2012). K5-R1/R2 mice have reduced expression of tight junction proteins, such as claudins 3 and 8 and occludin, leading to tight junction abnormalities and a consequent defect in the inside-out barrier of the epidermis (Yang et al., 2010). In addition, they also show increased expression of Sprrs (this study). The combination of these abnormalities in the triple mutant mice therefore aggravates the hyperkeratotic phenotype. It seems likely that the increase in the viable epidermis and the stratum corneum is a compensatory response to partially restore barrier function.

Besides the strong disturbance of epidermal structure and keratinocyte differentiation, we detected more epidermal cells with DNA damage and more apoptotic cells in K5-R1/R1/caNrf2 mice than in the other mouse groups. This finding demonstrates that the cytoprotective effect of activated Nrf2 does not prevent this damage, possibly because of other alterations that are not directly controlled by Nrf2, e.g. the loss of tissue integrity. The increase in apoptotic cells was compensated by a strong increase in poorly differentiated keratinocytes, which may reflect a more rapid epidermal turnover that does not allow enough time for proper keratinocyte differentiation. This provides a likely explanation for the reduction in senescent cells and also in epidermal T cells. T cells in the murine epidermis are almost exclusively γδ T cells, which exert potent anti-tumorigenic functions (Macleod and Havran, 2011). Their strong reduction in mice with activated Nrf2 in keratinocytes is consistent with the previously demonstrated oncogenic activity of activated Nrf2 in these cells (Rolfs et al., 2015).

In contrast to the strong epidermal phenotype of K5-R1/R2/caNrf2 mice, transient and milder activation of endogenous Nrf2 in the skin did not have the same deleterious consequences. Furthermore, activation of Nrf2 in keratinocytes of adult mice may be less harmful than the chronic activation of the Nrf2 pathway that is achieved in K5-caNrf2 mice during prenatal and postnatal skin development. This suggests that short-term application of NRF2-activating compounds to AD patients may also be tolerable, in particular when the doses are not too high. NRF2-activating compounds have already been approved for the treatment of other diseases and were shown to be safe and efficient: dimethyl fumarate (Tecfidera^®^) is an oral medication approved for the treatment of remitting multiple sclerosis (Venci and Gandhi, 2013; Blair, 2019); fumaric acid esters (Fumaderm^®^) are used for the treatment of moderate to severe psoriasis and are also administered orally (Walker et al., 2014). Therefore, they may also be suitable for use in AD. However, it remains to be determined whether the reduced NRF2 activity in the epidermis of AD patients (Koch et al., 2023) limits the efficacy of these compounds as seen in our mouse model.

Taken together, the results obtained in this study show that either loss or constitutive activation of Nrf2 in an AD-like mouse model aggravates the phenotype by interference with normal keratinocyte differentiation and, most likely, by increasing barrier dysfunction and cell damage. Restricted pharmacological activation of Nrf2, however, moderately improved epidermal barrier function. These findings demonstrate that the extent and duration of NRF2 activation in AD patients should be tightly controlled because of the multiple activities of NRF2 that affect epidermal homeostasis.

## MATERIALS AND METHODS

### Animal experiments

Mice were housed under specific pathogen-free conditions at the ETH Zurich Phenomics Center in a 12 h dark/light cycle with water and food *ad libitum*. All animal experiments were conducted under previous or current animal experiment licenses (to S.W.), approved by the local veterinary authorities (Cantonal Veterinary Office, Zurich, Switzerland).

Mice with conditional knockout of *Fgfr1* and *Fgfr2* in keratinocytes (K5-R1/R2 mice) had been generated by mating of mice with floxed *Fgfr1* and *Fgfr2* alleles with transgenic mice expressing Cre recombinase under control of the keratin 5 (K5) promoter (Yang et al., 2010). Mice with a conditional knockout of *Nrf2* in keratinocytes (K5-Nrf2 mice) or with Cre-inducible expression of the *caNrf2* transgene were also previously described (Schäfer et al., 2012; Telorack et al., 2016). To generate mice with triple knockout of *Fgfr1*, *Fgfr2* and *Nrf2* in keratinocytes, we crossed K5-R1/R2 mice with K5-Nrf2 mice. To generate mice with conditional knockout of *Fgfr1* and *Fgfr2* that express *caNrf2* in keratinocytes, we crossed K5-R1/R2 mice with K5-caNrf2 mice. Animals were genotyped using genomic DNA extracted from ear clip biopsies. DNA was amplified by PCR using the KAPA2G FAST Genotyping Mix (Kapa Biosystems, Wilmington, MA, USA) and analyzed by agarose gel electrophoresis. Genotyping primers are listed in Table S1.

To pharmacologically activate endogenous Nrf2, control and K5-R1/R2 mice were treated topically with DL-sulforaphane (SFN; Merck, Darmstadt, Germany). SFN was dissolved in DMSO (Merck) and mixed with hydrophilic non-ionic cream (Formula magistralis, Bahnhof Apotheke Zurich, Switzerland) at a final concentration of 10 mM. Twelve-week-old control and K5-R1/R2 mice were weighed and anesthetized with isofluorane (Rothacher-Medical, Heitenried, Switzerland). The back skin was shaved using an Aesculap clipper Isis (Braun, Melsungen, Germany), and 8 mg cream containing either SFN or DMSO only (vehicle) was applied to the back using a cotton swab. Mice were treated twice per week for 4 weeks, and back skin was harvested 6 h after the last treatment.

### Measurement of TEWL

TEWL was measured using a Tewameter^®^ TM 300 (Courage and Khazaka, Cologne, Germany). Measurements were taken at two different locations on the mouse back (left and right) with ten measurements each. The average of the 20 measurements was used for analysis.

### Isolation of primary murine keratinocytes

Primary murine keratinocytes were isolated from 3- to 5-day-old pups as described previously (Rauschendorfer et al., 2021), with the following modifications: cells were cultivated in CnT-07 Epithelial Proliferation Medium (CELLnTEC Advanced Cell Systems, Berne, Switzerland), which was supplemented with CnT-IsoBoost (CELLnTEC Advanced Cell Systems) for the first 2 days, and further maintained for up to 1 week.

### Epidermis–dermis separation, RNA isolation and RT-qPCR analysis

Separation of epidermis from the underlying dermis was achieved by heat shock treatment of mouse back skin by incubating the skin for 30 s at 60°C, followed by 1 min at 4°C, both in DEPC-PBS. For RNA isolation from tissue, the epidermis was placed in 1 ml Trizol (Thermo Fisher Scientific, Waltham, MA, USA). The epidermis was gently peeled off using forceps and homogenized using an IKA T25 ULTRA-TURRAX Disperser (IKA Labortechnik, Staufen, Germany). Samples were incubated at room temperature (RT) for 5 min. Then, 200 μl chloroform was added to each sample, and samples were left for extraction for 10 min. After centrifugation at 16,200 ***g*** for 15 min at 4°C, the upper (aqueous) layer of ∼500 μl was transferred to a new 2 ml tube, 500 μl chloroform was added, and the samples were mixed again. After 10 min, samples were centrifuged again as described above. The supernatant (∼500 μl) was carefully mixed with 500 μl isopropanol and stored at −20°C overnight (o/n) to allow for precipitation. Next, samples were centrifuged at 13,800 ***g*** for 8 min at 4°C. The RNA pellet was washed twice with 70% ethanol and subsequently centrifuged at 5400 ***g*** for 5 min at 4°C. The extracted RNA was dissolved in 25 μl DEPC-H_2_O. For RNA isolation, cells were lysed in 350 μl RB lysis buffer (IBI Scientific, Dubuque, IO, USA). RNA isolation was performed using a Mini Total Tissue RNA kit (IBI Scientific), including a DNase digestion step, and the RNA was dissolved in 30 μl DEPC-H_2_O according to the manufacturer's protocol.

Reverse transcription of 1 μg RNA was performed using an iScript cDNA synthesis kit (Bio-Rad) according to the manufacturer's protocol. cDNA was diluted 1:10 (cells) or 1:5 (tissue), and 5 μl cDNA was mixed with 5.5 μl LightCycler SYBR Green (Roche, Basel, Switzerland) and 0.5 μl primer mix (10 μM; Microsynth, Balgach, Switzerland). Relative gene expression was determined using a LightCycler 480 II (Roche). Relative expression levels were calculated using the 2^−ΔΔCt^ method (Livak and Schmittgen, 2001), and gene expression was normalized to the expression of the housekeeping gene *Rps29*. RT-qPCR primers are listed in Table S2.

### Corneocyte stability assay

Corneocyte isolation and determination of their stability was assessed as described previously (Schäfer et al., 2012). Cells were counted using a Neubauer chamber, and 500,000 corneocytes/ml in 2% sodium dodecyl sulfate were sonicated using a Bioruptor^®^ Pico sonication device (Diagenode, Seraing, Belgium) at 4°C; an aliquot was taken after every minute of sonication. Afterwards, cell solutions were imaged using a LUNA-II™ Automated Cell Counter (Logos Biosystems, Anyang, South Korea), and the number of intact corneocytes was counted manually.

### Histological and immunological staining

Samples were fixed in 4% paraformaldehyde (PFA) or acetic ethanol, processed and sectioned. H&E staining (Rauschendorfer et al., 2021) was performed on 7 µm paraffin sections of back skin fixed o/n in 4% PFA. Toluidine Blue staining was performed on acetic ethanol-fixed paraffin-embedded 7 µm sections of back skin (Sulcova et al., 2015; Yang et al., 2010).

For immunofluorescence on paraffin sections (γH2AX), slides were dewaxed and rehydrated using a xylene/ethanol gradient. Antigen retrieval was performed in citrate buffer (100 mmol/l citric acid, pH 6.0) at 95°C for 1 h, followed by three washes with PBS. For immunofluorescence on cryosections (cleaved caspase 3), slides were briefly air dried, fixed in methanol for 10 min at −20°C, and then washed three times for 5 min each in PBS with 0.05% Tween 20 (TBS-T).

Subsequently, non-specific binding sites were blocked by incubating the sections in 12% bovine serum albumin (BSA), 0.1% NP40 in PBS at RT for at least 1 h. Sections were then incubated with the primary antibody o/n at 4°C, diluted in 5% BSA, 0.03% NP40 in PBS, except for the anti-Ki67 antibody, which was incubated for 15 min at RT. The next day, the primary antibody was removed, and sections were washed three times for 15 min each with PBS and incubated with the appropriate secondary antibody diluted in 5% BSA, 0.03% NP40 in PBS, along with Hoechst nuclear dye (1:500, Molecular Probes, Eugene, OR, USA) for 1 h at RT. Finally, sections were washed three times for 5 min each with PBS, rinsed with H_2_O, and coverslipped using Mowiol containing 10% DABCO (both from Sigma-Aldrich, St. Louis, MO, USA). Sections were air dried o/n and imaged the following day.

For immunohistochemistry on paraffin-embedded sections, slides were dewaxed and rehydrated using a xylene/ethanol gradient. Sections were washed three times in TBS and incubated in 3% H_2_O_2_ in methanol at RT for 15 min to block endogenous peroxidases. Tissues were incubated in 100 mM citrate buffer for 1 h, and blocking and primary antibody incubation were performed as described above. The next day, the primary antibody was removed, and sections were washed three times for 15 min each with TBS and incubated with the respective secondary antibody, diluted in 4% BSA, 0.03% NP40 in TBS, for 1 h at RT. After washing the sections three times for 15 min with TBS, they were incubated with a VECTASTAIN^®^ Elite^®^ ABC-HRP Kit (Vector Laboratories, Burlingame, CA, USA) for 30 min. Slides were then washed twice for 5 min with TBS, and DAB substrate (Vector Laboratories) was applied for ∼5 min. The reaction was stopped by rinsing the slides with H_2_O, and the sections were counterstained with Hematoxylin. Finally, they were dehydrated using an increasing ethanol gradient, incubated twice for 10 min in xylene, briefly air dried and coverslipped using Eukitt mounting medium (Merck). All used antibodies are listed in Table S3.

Immunofluorescence images were acquired using one of the following platforms: Zeiss Imager.A1 microscope equipped with enhanced-contrast Plan-Neofluar objectives (40×/1.3 NA oil and 20×/0.5NA) equipped with an AxioCam MRm camera (all from Carl Zeiss, Oberkochen, Germany) or Zeiss Axioscan 7, equipped with an Axiocam 712 mono camera (both from Carl Zeiss). Brightfield images were acquired using the Zeiss Axioscan 7, equipped with an Axiocam 705 color camera (Carl Zeiss). Images on all platforms were acquired using ZEN Blue software. All images were exported and processed, if necessary, using FIJI (National Institutes of Health, Bethesda, MD, USA) and/or QuPath (Bankhead et al., 2017).

### β-Galactosidase staining

For β-galactosidase staining, the β-galactosidase staining solution was freshly prepared each time. Cryosections were cut, and each slide was processed separately and immediately before the staining solution was applied to minimize drying of sections. Sections were washed for 1 min in PBS, fixed for 1 min in 1% PFA in PBS and washed twice for 1 min in PBS. They were incubated in β-galactosidase staining solution [40 mM citric acid/Na phosphate buffer, 5 mM K_4_FE(CN)_6_×3 H_2_O, 5 mM K_3_FE(CN)_6_, 150 mM NaCl, 2 mM Mg/Cl_2_, 1 mg/ml X-Gal (AppliChem, Darmstadt, Germany), pH 5.5] for 48 h at 37°C without CO_2_. Afterwards, sections were washed for 1 min in PBS, incubated for 1 min in methanol, washed for 1 min in PBS, counterstained with Eosin as described above and coverslipped using Eukitt. Imaging was performed as described above.

### Statistical analysis

Statistical analysis was performed using Prism (v9; GraphPad Software, San Diego, CA, USA). For statistical testing of two unmatched groups, Mann–Whitney test was used as a normal distribution could not be assumed at the given sample numbers. For statistical testing of three or more unmatched groups, one-way ANOVA was used. To analyze the effects of two categorical independent variables on a continuous dependent variable, two-way ANOVA was used. *P*<0.05 was considered significant.

## Supplementary Material

10.1242/dmm.052126_sup1 Supplementary information
