# Supplementary material for: Dose-dependent effects of Nrf2 on the epidermis in chronic skin inflammation
Source: Dis Model Mech. 2025 Jan 2;18(1):dmm052126. doi: 10.1242/dmm.052126 (PMC11708820; doi:10.1242/dmm.052126)
Supplement: Supplementary information [file dmm-18-052126-s1.pdf]

A

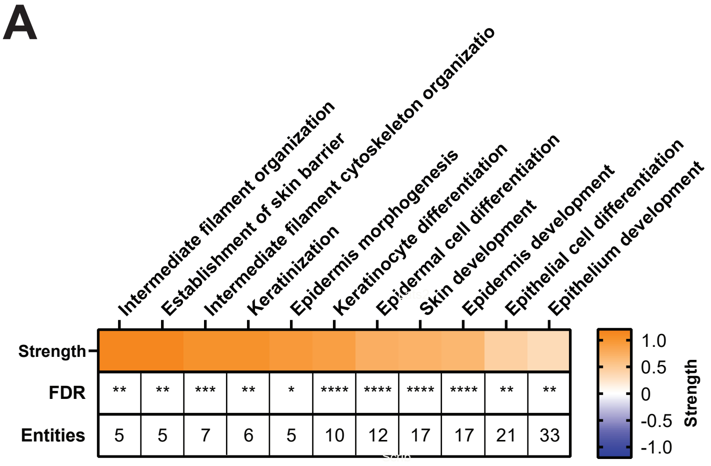

C

	Il-1 Signaling	Il-4 and Il-13 Signaling	Il-3, Il-5 and GM-CSF Signaling	Il-17 Signaling	IFNγ Signaling
**P-value**	**	n.s.	n.s.	n.s.	n.s.
**Entities**	28	1	4	4	1
**Reactions**	11	11	20	8	1

B

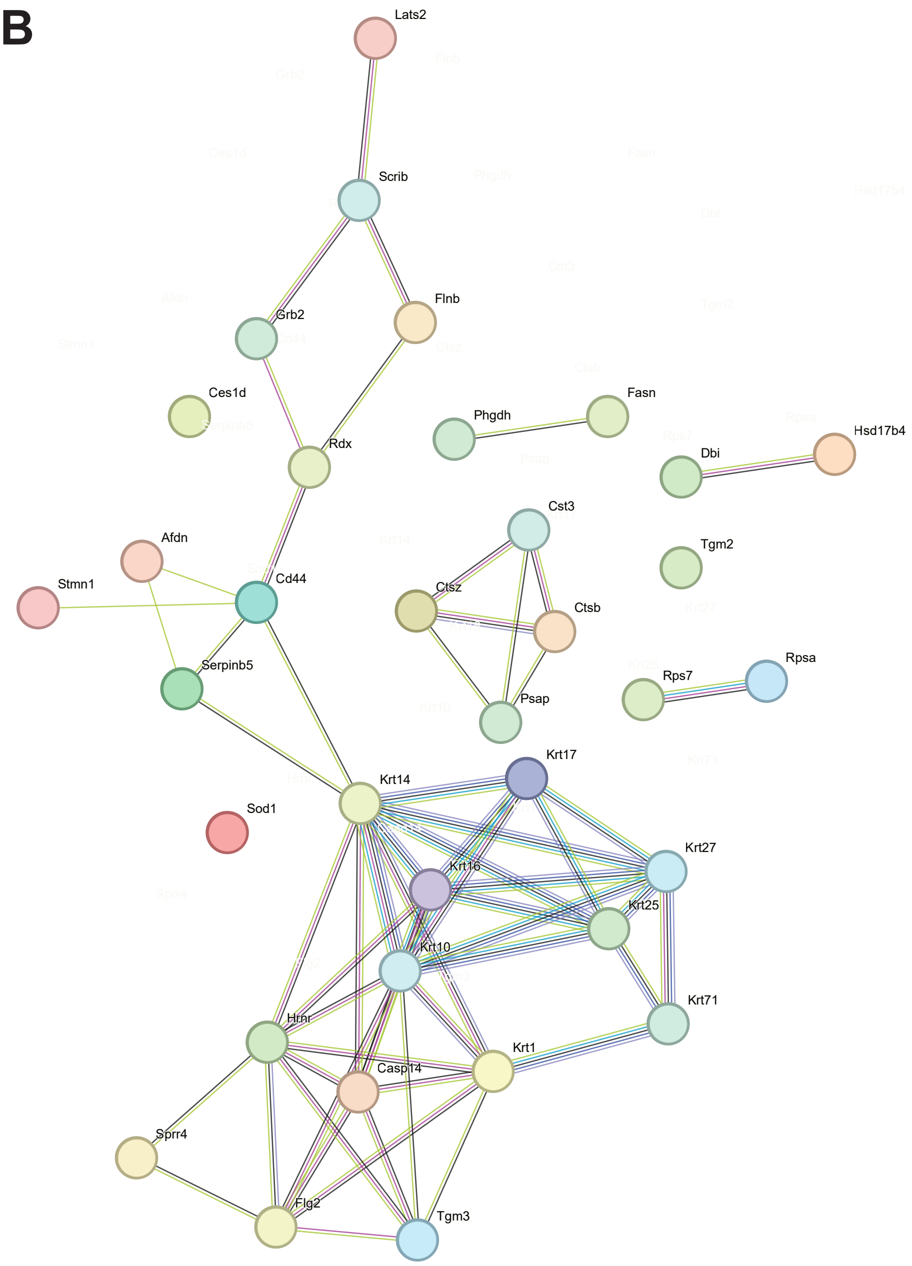

D

Pro-inflammatory mediators

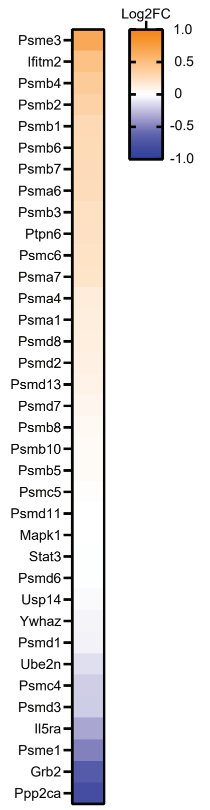

Disease Models & Mechanisms • Supplementary information

Fig. S1. Proteomics analysis reveals perturbed epidermal differentiation in K5-R1/R2 mice
(**A**) Heatmap of altered pathways related to epidermal development, differentiation and barrier function based on STRING analysis (Szklarczyk et al., 2019) of the epidermal proteome of K5-R1/R2 and control (R1/R2) mice. Strength of regulation, false discovery rate (FDR) and number of detected entities (proteins) are shown. Proteins exhibiting a Log2FC < -0.58 or >0.58 were included in the analysis. *FDR < 0.05, **FDR < 0.01, ***FDR < 0.001, ****FDR < 0.0001.
(**B**) STRING analysis of all differentially abundant proteins involved in the pathways listed in (A). Edge colors indicate: from curated database (turquoise), experimentally determined (pink), gene neighborhood (green), gene fusions (red), gene co-occurrence (blue), textmining (light green), co-expression (black), and protein homology (light blue).
(**C**) Table showing the regulation of pro-inflammatory pathways in the epidermis of K5-R1/R2 *vs.* control mice. Functional analysis was performed using ProteoRe.org. n.s. = non-significant, **p < 0.01.
(**D**) Heat map showing individual differentially abundant proteins of the pathways listed in (C).

Disease Models & Mechanisms • Supplementary information

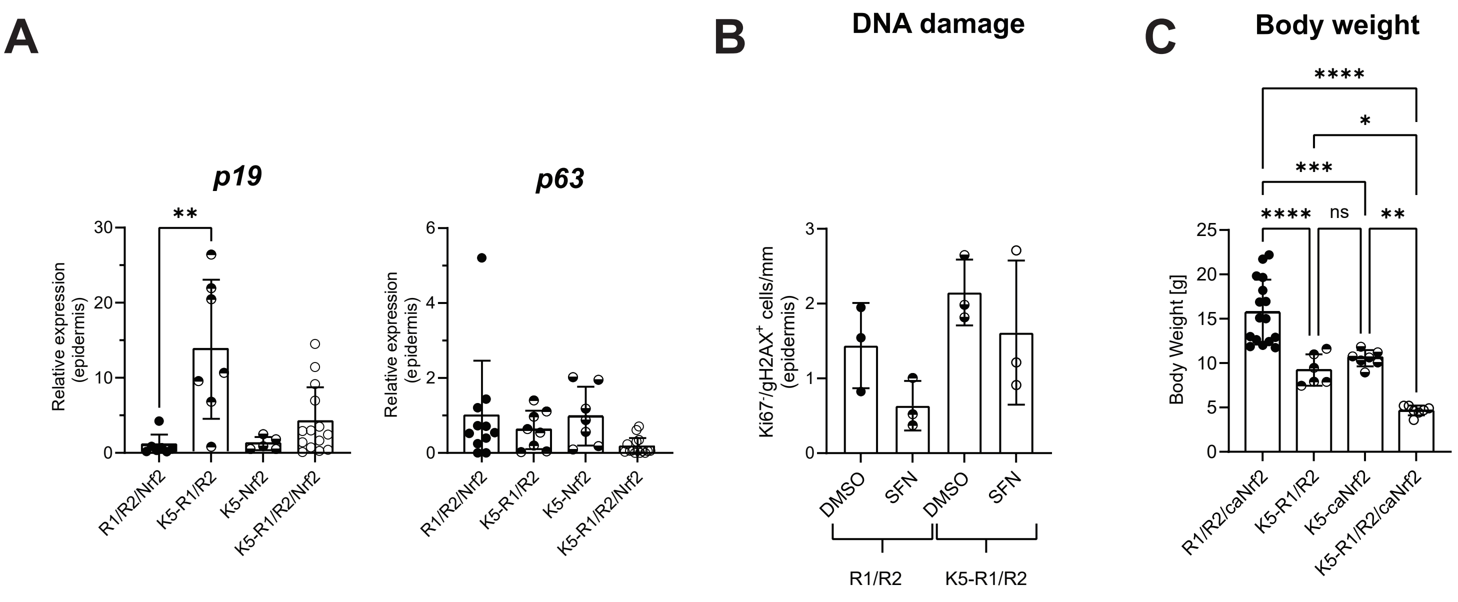

Fig. S2. Additional features of K5-R1/R2/Nrf2 mice and of SFN-treated K5-R1/R2 mice.

(**A**) RT-qPCR for *p63* and *p19* relative to *Rps29* using RNA from the epidermis of control, K5-R1/R2, K5-Nrf2 and K5-R1/R2/Nrf2 mice at 6 weeks of age. N = 5-14 mice per genotype.

(**B**) Quantification of epidermal $Ki67^-/\gamma H2AX^+$ keratinocytes in control and K5-R1/R2 mice after a four-week treatment with SFN or vehicle. N = 3 mice per genotype and treatment group. See Fig. 3G for DNA damage in a second SFN treatment experiment.

(**C**) Body weight of control, K5-R1/R2, K5-caNrf2 and K5-R1/R2/caNrf2 mice at 3 weeks of age. N = 7-16 mice per genotype. **Graphs indicate mean and standard deviation. n.s. = non-significant, *p < 0.05, **p < 0.01, ***p < 0.001, ****p < 0.0001 (One-way ANOVA).**

Disease Models & Mechanisms • Supplementary information

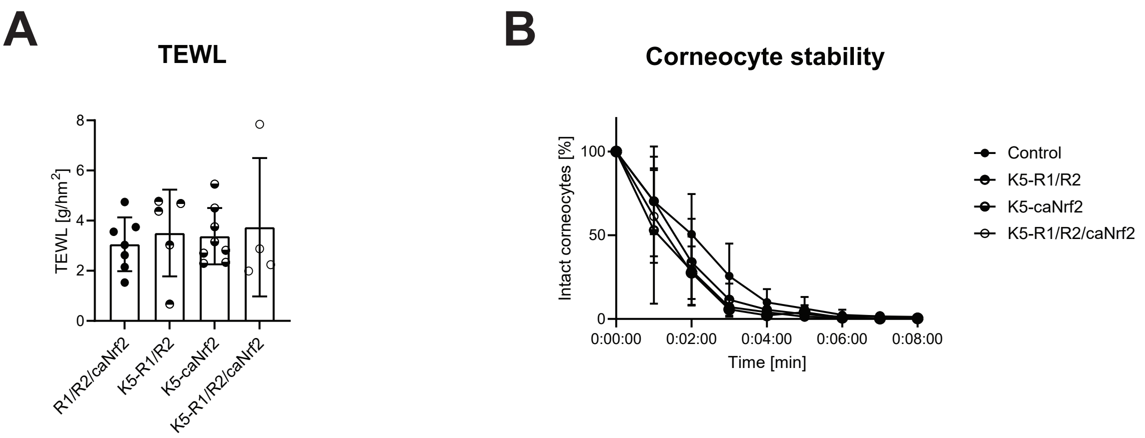

Fig. S3. Neonatal K5-R1/R2/caNrf2 mice do not exhibit epidermal abnormalities. (**A**) Quantification of transepidermal water loss (TEWL) of neonatal control, K5-R1/R2, K5-caNrf2 and K5-R1/R2/caNrf2 mice. N = 4-8 mice per genotype.

(**B**) Quantification of the percentage of intact freshly isolated corneocytes from neonatal control, K5-R1/R2, K5-caNrf2 and K5-R1/R2/caNrf2 mice at different time points of sonication. N = 4-8 mice per genotype.

Table S1. Genotyping primer sequences

Gene	Forward Primer	Reverse Primer
Fgfr1	CGAATGGACAAGCCCAGTAAC	CTCCTGCTTCCTTCAGAGC
Fgfr2	ATAGGAGCAACAGGCGG	TGCAAGAGGCGACCAGTCAG
K5-Cre	AACATGCTTCATCGTCGG	TTCGGATCATCAGCTACACC
Nrf2	TCTTAGGCACCATTTGGGAGAG	TACAGCAGGCATACCATTGTGG
caNrf2	CGGCTCAGCACCTTGTAT	CTTATTCCAAGCGGCTTCGGC

Table S2. RT-qPCR primer sequences

Gene	Forward Primer	Reverse Primer
Gclc	AACAAGAAACATCCGGCATC	CGTAGCCTCGGTAAAATGGA
Nqo1	CTGGCCCATTCAGAGAAGAC	GTCTGCAGCTTCCAGCTTCT
Nrf2	CCAGCTACTCCCAGGTTGC	CCAAACTTGCTCCATGTCCT
p19	TGGAGCAGAAGAGCTGCTACGT	GCCGCACCGGAATCCT
p63	CCCAGTCATCTGATTCGAGT	GTTCTTTGCGCTGTCCGATA
Rps29	GGTCACCAGCAGCTCTACTG	GTCCAACTTAATGAAGCCTATGTCC
Sprr2a	GAACCTGATTCTGAGACTCAA	GCACACTACAGGACGACAC
Sprr2d	CTGGTACTCAAGGCCGAGAC	CAGGGCACTTTGGTGGAG
Sprr2h	GACACTTGGTACTCAAGCTCTGG	TGCACTGCTGCTGTTGGTAA

Table S3. Primary and secondary antibodies used for immunostaining

Primary antibodies			
Antibody	Cat. No.	Manufacturer	Dilution
Biotin anti-γH2Ax (Ser139)	16-193	Merck, Darmstadt, Germany	1:300
Rabbit anti-Ki67	ab15580	Abcam, Cambridge, UK	1:200
Rabbit anti-loricrin	19051	BioLegend, San Diego, CA	1:500
Rabbit anti-KRT10	PRB-159P	BioLegend	1:500
Rabbit anti-KRT14	PRB-155P	BAbCo, Richmond, CA	1:1000
Rabbit anti-cleaved caspase 3	9661	Cell Signaling Technology, Danvers, MA	1:100
Rabbit anti-CD3	A0452	Agilent Dako, Santa Clara, CA	1:200
Rat anti-Ly6G	551459	BD Biosciences, Franklin Lakes, NJ	1:100
**Secondary antibodies**			
Streptavidin-Cy3	405215	BioLegend	1:500
Goat anti-rabbit IgG–Cy3	111-165-003	Jackson ImmunoResearch Laboratories, Westgrove, PA	1:200
Goat anti-rabbit biotin	111-065-003	Jackson ImmunoResearch Laboratories	1:1000
Rabbit anti-rat biotin	BA-4001	Vector Laboratories, Inc.	1:1000
Donkey anti-rabbit IgG Alexa 488	71-547-003	Jackson ImmunoResearch Laboratories	1:400

Disease Models & Mechanisms • Supplementary information
